# Enzymatic Modification of Pomace Olive Oil with Natural Antioxidants: Effect on Oxidative Stability

**DOI:** 10.3390/biom13071034

**Published:** 2023-06-23

**Authors:** Renia Fotiadou, Dimitrios Lefas, Despina Vougiouklaki, Aliki Tsakni, Dimitra Houhoula, Haralambos Stamatis

**Affiliations:** 1 Laboratory of Biotechnology, Department of Biological Applications and Technologies, University of Ioannina, 45110 Ioannina, Greece; renia.fotiadou@gmail.com (R.F.); bl02000@uoi.gr (D.L.); 2 Department of Food Science and Technology, University of West Attica, 12243 Athens, Greece; dvougiouklaki@hotmail.com (D.V.); aliki_tsak@yahoo.gr (A.T.); dhouhoula@uniwa.gr (D.H.)

**Keywords:** phenolipids, hydrolase, *Thermomyces lanuginosus* lipase, immobilized lipase, green nanoparticles, acylation, reusability, bioactive compounds, hydroxytyrosol, mayonnaise

## Abstract

Enzymatic lipophilization has been proposed as a cost-effective strategy to produce new liposoluble antioxidant compounds. In this study, modified oils rich in structured phenolipids were prepared via one-pot enzymatic acylation of hydroxytyrosol (HTYR), vanillyl alcohol (VA) and homovanillyl alcohol (HVA) with pomace olive oil (POO) in solvent-free conditions using immobilized lipase on biogenic nanoparticles. The effect of temperature (30–70 °C) and enzyme concentration (0.1–1%, *w*/*w*) on the efficiency of the bioprocess as well as the reusability of the nanobiocatalyst were thoroughly investigated. The modified oils exhibited increased antioxidant activity compared to the control oil according to DPPH and CUPRAC assays (*p* < 0.05). The oxidative stability of pomace olive oil was also significantly enhanced after modification, as depicted by the *K_232_* values and TBARS contents under accelerated oxidation at 60 °C (*p* < 0.05). Moreover, a fortified mayonnaise containing modified oil with HTYR was prepared that was noticeably stable compared to the control mayonnaise at 28 °C for 5 months (*p* < 0.05). Enzymatically modified oils have great potential for application in the nutraceutical and food industry, encouraging the exploitation of immobilized lipases as effective and green catalytic tools.

## 1. Introduction

Natural phenolic compounds display several biological activities, such as antiradical/antioxidant, antibacterial, etc. Concerning the increasing trend for natural ingredients in nutraceutical and food industries driven by the consumer demand, the research on natural polyphenol effects is rising. Hydroxytyrosol (HTYR), vanillyl alcohol (VA) and homovanillyl alcohol (HVA) are among the major phenolic compounds found in the by-products of the olive oil industry [1,2]. HTYR and VA exhibit antioxidant, anti-inflammatory and antibacterial activities [2,3,4], while HVA possesses antioxidant and antiradical activity and is considered as a more stable compound in biological fluids [5]. Furthermore, HTYR is identified as one of the most potent natural antioxidants recovered from agro-industrial wastes and safe for human consumption according to the European Food Safety Authority (EFSA) [6]. However, the poor solubility of these natural polyphenols in lipid media limits their application due to reduced biological activity [2,7]. 

Pomace olive oil (POO) is also a by-product of the downstream process of the olive oil industry. It is cheap and finds several commercial applications in cosmetics, nutraceuticals, and food products. The high monounsaturated fatty acid ratio and specific phytosterols account for its proven biological activities such as the cardioprotective. Moreover, it contains an adequate level of linoleic acid which is an essential fatty acid [8,9,10]. However, the mono- and polyunsaturated fatty acids (MUFAs, PUFAs) of POO are prone to autoxidation. Autoxidation is a result of the spontaneous reaction of molecular oxygen with fatty acids which contain one or more double bonds. Environmental (extrinsic) and intrinsic factors, such as temperature, light, exposure to oxygen, metals, the profile of fatty acids and other chemical compounds in the oil, affect or initiate the autoxidation which results in precocious rancidity. Rancid oils are characterized by poor taste, smell, texture, change of color, and production of prospective toxic or carcinogenetic organic molecules [7,11,12]. It is well documented that the addition of antioxidants moderates the cascade reaction of autoxidation. For many years, the incorporation of synthetic lipophilic antioxidants as preservatives in food matrices has been applied. However, the ongoing need of consumers for natural products brings the replacement of synthetic antioxidants in implementation. Hence, the preparation of novel structured phenolipids is now of high interest. Lipophilic derivatives of natural polyphenols could exert a protective effect by enhancing the oxidative stability of oils and thus delaying the onset of rancidity [7,13]. 

The lipophilization of phenolic compounds with different acyl donors has been presented as a strategy in order to produce lipophilic antioxidants [2,7]. Esterification of the primary alcohol group of phenolics with different fatty acids does not impair their phenolic moiety which is responsible for their antioxidant activity. Enzymatic acylation is preferred over chemical synthesis due to more friendly, economical, and benign reaction conditions which allow the recovery of pure products and the reuse of the biocatalyst [14]. Free fatty acids and their methyl, ethyl and vinyl esters have been applied as acyl donors [15]. Recently, our laboratory has reported the enzymatic synthesis of tyrosol and HTYR esters with different fatty acids in organic solvent systems (OSS) using novel immobilized lipases as biocatalysts [16,17]. However, the utilization of edible oils seems a cheaper alternative and a more abundant and natural source of different fatty acids. For instance, Sun et al. reported the production of medium-chain HTYR esters using castor oil, and Natalia et al. highlighted the synthesis of VA esters using menhaden oil in OSS [4,18]. A significant drawback of these approaches is the use of organic solvents, which limits or prevents the applicability of these products in foods and nutraceuticals and increases the overall operating costs. Edible oils can serve as reaction media for lipase-catalyzed reactions containing high levels of MUFAs and PUFAs to avoid OSS. For instance, Zhang et al. reported the preparation of a “functional oil” rich in feruloylated structured lipids with solvent-free ultrasound pre-treatment [19].

In the current work, we report the biocatalytic synthesis of novel lipophilic compounds using a newly developed nanobiocatalytic system of immobilized lipase from *Thermomyces lanuginosus* (TLL) in solvent-free conditions (SFS) aiming to increase the oxidative stability of a common vegetable oil. The present study highlights the application of POO both as reaction medium and acylating agent in a one-pot reaction system to acquire a final modified oil rich in structured phenolipids. Three different phenolic alcohols (HTYR, VA and HVA) were tested as substrates while POO served as the only acyl donor. The kinetics of reactions was investigated, and the products were characterized by High-Performance Liquid Chromatography (HPLC) and Mass Spectroscopy (MS). Various reaction parameters which affect the effectiveness of the biocatalytic process such as the temperature, the enzyme concentration, and the reusability of the nanobiocatalyst were thoroughly evaluated. The antioxidant activity and the oxidative stability of the novel oils-enriched with lipophilic derivatives of natural antioxidants were investigated using a combination of various techniques. To the best of our knowledge, a systematic study on the antioxidant activity and the oxidative stability of modified oils with natural antioxidants has not been reported before. Furthermore, the modified oil with HTYR was exploited as a constituent of mayonnaise sauce, and the oxidative stability of the formulation was tested against a control sample.

## 2. Materials and Methods

### 2.1. Materials

Hydroxytyrosol, vanillyl alcohol and homovanillyl alcohol (>98%) were purchased from Carbosynth (Compton, Berkshire, UK). Pomace olive oil was obtained from the local supermarket. Lipase from *Thermomyces lanuginosus* was kindly provided by Novozymes A/S (Bagsvaerd, Denmark). Methanol, acetonitrile, n-hexane, heptane, ethyl acetate, and water (HPLC grade) were purchased from Fisher Scientific Corporation (Loughborough, UK). All other reagents were of analytical grade, purchased from Sigma-Aldrich (St. Louis, MO, USA). 

### 2.2. Methods

#### 2.2.1. Immobilization of Lipase on Green Nanoparticles

Lipase from *Thermomyces lanuginosus* was immobilized on the surface of green synthesized zinc oxide-iron oxide nanoparticles (average size 15–17 nm) as previously described [16]. The nanobiocatalytic system of immobilized lipase (ZnOFe-TLL, average size 20–24 nm) was exploited for further experiments.

#### 2.2.2. Analysis of the Fatty Acid Composition of Pomace Olive Oil

The fatty acid composition of POO was elucidated by Gas Chromatography–Mass Spectrometry analysis (GC/MS). Methylated derivatives of fatty acids were prepared according to analytical methods described in the European Regulation [20] and analysed using a GC/MS system (GCMS-QP2010 SE, Shimadzu, Tokyo, Japan), equipped with a split/splitless injector, a simple quadrupole mass spectrometer operating in electronic ionisation (EI) mode (70 eV) and a MEGA-5 MS capillary column (30 m length × 0.25 mm internal diameter × 0.25 μm film thickness; MEGA, Legnano, Italy). The oven was programmed from 150 °C (hold 1 min) to 250 °C (hold 5 min) with a heating rate of 3 °C min^−1^. The carrier gas (helium) was set to a flow rate of 1.4 mL min^−1^. The injector was operated in a split mode (1:25) at 270 °C. The mass spectrometer was set at the following conditions: ion source temperature at 150 °C and interface (transfer-line temperature) at 250 °C; it was operated in a scan mode (*m*/*z* 35–450). The identification of fatty acid methyl esters (FAMEs) was based on the NIST library and the retention times of standard compounds.

#### 2.2.3. Enzymatic Preparation of Modified Oils 

The enzymatic acylation of natural antioxidants was performed in round-bottom flasks under nitrogen, containing POO and 0.3% (*w*/*w*) of the phenolic compound (HTYR, VA, HVA) in a solvent-free system. The reactions were initiated by adding 1% (*w*/*w*) of the immobilized lipase (ZnOFe-TLL), and incubated at 50 °C under orbital shaking and absence of light. Aliquots (50 μL) were withdrawn at specified time intervals (0, 1, 2, 3, 4, 6, 24 h) and the progress of reactions was monitored by High-Performance Liquid Chromatography (HPLC) coupled with a Photodiode Array Detector (PDA). All the reactions were conducted in triplicate. Samples without enzyme were also incubated and no products were detected.

##### Effect of Temperature and Enzyme Concentration

The enzymatic acylation of POO with HTYR was carried out under different temperatures (30–70 °C) and enzyme concentrations (0.1–1%, *w*/*w*) to determine the optimal conditions for the preparation of the modified oil. The reactions were carried out as described in Section 2.2.3. After 24 h of incubation, samples were withdrawn and monitored by HPLC-PDA. All the reactions were conducted in triplicate.

##### Reusability of the Nanobiocatalytic System

The reusability of the immobilized lipase for the preparation of the modified oil with HTYR was evaluated in the optimal conditions. After each cycle, the nanobiocatalytic system was recovered using an external magnetic force, washed with tert-butyl methyl ether, and subsequently applied to a fresh reaction solution for ten successive reaction cycles (240 h of total operation). The conversion yield of the first cycle was taken as the control for the calculation of the residual activity after each use. All the reactions were conducted in triplicate.

#### 2.2.4. Characterization and Quantification of the Products

##### High-Performance Liquid Chromatography Analysis

The reaction aliquots were mixed with methanol (1:5) using vigorous shaking. Then, the mixtures were centrifugated at 12,000 for 5 min. The methanol phases were recovered and filtrated using Nylon Syringe filters (0.22 μm, 13 mm). HPLC analysis of the different reaction samples was carried out as previously reported [16]. Moreover, the main products of its reaction were isolated by semi-preparative HPLC as described before. Standard curves of the natural antioxidants and their acylated derivatives were prepared in methanol. The conversion and production yields were calculated as described previously [16,21,22]. The absorption maximum of the initial substrates and products was the same.

(1) $$C_{substrate}\left(mM\right)=\frac{A_{substrate}}{Slope}\times \;\mathrm{DF},\;$$

(2) $$C_{product\;}\left(mM\right)=\frac{A_{product}}{Slope^{\prime }}\times \;\mathrm{DF},\;$$

where C*_substrate_* and C*_product_* are the concentrations (mM) of substrate that remained at t_x_ of reaction and the concentration of its product synthesized at t_x_ of reaction, respectively. A*_substrate_* and A*_product_* are the respective areas at t_x_ of reaction, and DF is the dilution factor. Experiments were performed in triplicate.

##### Mass Spectrometry Analysis

Mass Spectrometry (MS) analysis was carried out using a Mass Spectrometer Advion system equipped with an atmospheric pressure chemical ionization (APCI) interface according to [23,24] with some modifications. The mass analyser is a single quadrupole. The quadrupole consists of four parallel rods that use electric fields to filter ions based on the *m*/*z* ratio. The type of detector which is used is an electron amplifier. The conditions used for Mass Spectrometry for APCI positive source were: nebulizing gas (N_2_) flow rate 1.5 L min^−1^, Capillary Voltage 180 V, Source Voltage Offset 25 V, Source Voltage Span 20 V, Source Gas Temperature 350 °C, Capillary Temperature 200 °C and APCI Corona Discharge 5 μA. A volume of 10 μL of the sample was directly injected. Samples were dissolved in methanol. For full-scan MS analysis, the spectra were recorded in the positive mode in the range of *m*/*z* 100–1000.

#### 2.2.5. Antioxidant Activity Tests

The 1,1-pheny-2-picrylhydrazyl (DPPH) and the Cupric Reducing Antioxidant Capacity (CUPRAC) assays were used to evaluate the antioxidant capacity of the unmodified and the modified oils. For the DPPH assay, different concentrations of oils were prepared in ethyl acetate. Appropriate quantities were added to 0.1 mM of DPPH methanolic solution to reach a final volume of 1 mL. The solutions were shaken vigorously and incubated for 30 min in the dark. The absorbance was measured at 517 nm using a 1 cm path length cell and a UV/VIS spectrophotometer (Agilent, Santa Clara, CA, USA). Methanol was used as a blank. A standard curve of Trolox (concentration range 2–30 μM) was used for quantification. The results were expressed as micromoles of Trolox equivalents per 100 g of oil. For the CUPRAC assay, different concentrations of oils were prepared in a 1:9 (*v*/*v*) water–acetone mixture. The procedure was adapted to a final volume of 287 μL and performed exactly as described by Özyürek et al. [25]. The absorbance was measured at 450 nm using a UV/Vis microplate reader (Multiskan SkyHigh, Thermo Fisher Scientific, Cleveland, OH, USA). Water–acetone mixture was used as a blank. A standard curve of Tocopherol (concentration range 2–80 μM) was used for quantification. The results were expressed as micromoles of Tocopherol equivalents per 100 g of oil. Experiments were performed in triplicate.

#### 2.2.6. Oxidative Stability Tests

##### Determination of Conjugated Dienes

Conjugated Dienes (CDs) formed at the primary stage of oxidation were measured spectroscopically according to analytical methods described in the European Regulation [20]. The absorbance was read at 232 nm using a 1 cm path length quartz cell and a UV/VIS spectrophotometer (Agilent, Santa Clara, CA, USA). Isooctane was used as a blank. CDs were assessed once per week for four weeks during the accelerated oxidation of oils at 60 °C in the dark. The results were expressed as *K_232_* values (specific extinction coefficient), which were calculated as follows:

(3) $$K_{232}=\frac{A_{232\mathrm{nm}}}{C\times l},$$

where *K_232_* is the specific extinction coefficient at *λ* = 232 nm, *A_232_* is the absorbance measured at *λ* = 232 nm, *C* is the concentration of the oil in isooctane (g 100 mL^−1^), and *l* is the light path of the cuvette (cm). Experiments were performed in triplicate.

##### Determination of Thiobarbituric Acid Reactive Substances 

Thiobarbituric Acid Reactive Substance (TBARS) assay detects the secondary oxidative products of lipid peroxidation. The assay was performed with slight modifications according to Zeb and Ullah [26]. In brief, 10 mg of each oil was added to 125 μL of 50% glacial acetic acid with double-distilled water (ddH_2_O). The homogenous solution was mixed with 125 μL of 1 mM Thiobarbituric acid (TBA) in 50% glacial acetic acid with ddH_2_O and incubated at 95 °C for 1 h. The absorbance was read at 532 nm using a UV/Vis microplate reader (Multiskan SkyHigh, Thermo Fisher Scientific, Cleveland, OH, USA). TBA solution in 50% glacial acetic acid with ddH_2_O was used as a blank. The TBARS were assessed once per week for four weeks during accelerated oxidation of oils at 60 °C in the dark. Malondialdehyde was used for the preparation of the standard curve (concentration range 0.25–25 μmol L^−1^). The results were expressed as micromoles of malondialdehyde equivalents per kg of oil according to the formula:

(4) $$TBARS\;\left(\frac{\mu mol}{kg}\right)=\frac{C\times W}{V},$$

where *C* is the concentration of malondialdehyde from the calibration curve (μmol L^−1^), 𝑉 is the total volume of the assay (L) and 𝑊 is the weight of the oil (kg). Experiments were performed in triplicate.

##### Determination of Volatile Oxidative Products

The analysis of the volatile compounds (secondary and tertiary oxidative degradation products) of oils was performed according to Beltran et al. [27] with minor modifications using Headspace Solid-Phase Microextraction coupled with the Gas Chromatography–Mass Spectrometry (HS-SPME-GC/MS) methodology. A divinylbenzene/carboxen/poly-dimethylsiloxane (DVB/CAR/PDMS) 50/30 μm, StableFlex, 1 cm, SPME fiber was used (Bipolar, Adsorbent, MW: 40–275, Supelco, Sigma Aldrich, St. Louis, MO, USA). Samples were incubated in 4 mL screw-capped vials with polytetrafluoroethylene/silicone septa at 60 °C in the dark. After a 5 min period of equilibration at 60 °C under stirring at 100 rpm, the SPME fiber was exposed to the vial headspace for 20 min. After that time, the SPME fiber was placed in the injection port of GC/MS for desorption (2 min). The SPME fiber was conditioned after every run at 250 °C for 30 min. 

The oven was programmed from 50 °C (hold 10 min) to 150 °C (hold 1 min) with a heating rate of 10 °C min^−1^. The carrier gas (helium) was set to a flow rate of 1 mL min^−1^. The injector was operated at 250 °C. The mass spectrometer was set at the following conditions: ion source temperature at 180 °C and interface (transfer-line temperature) at 200 °C, operated in a scan mode (*m*/*z* 40–500). The identification of volatile compounds was based on the NIST library (similarity index > 90%). The SPME-GC/MS analysis of oils was performed once per week for three weeks during accelerated oxidation at 60 °C in the dark. The results were expressed as relative peak area increase in comparison to an internal standard compound as described previously [27,28]. Experiments were performed in duplicate.

##### Fluorescence Spectroscopy 

A luminescence spectrofluorometer Jasco-8300 (Tokyo, Japan) was used for the assessment of the oxidative adulteration of oils. The fluorescence emission spectra were recorded from 600 to 750 nm after excitation of the oil samples at 400 nm, with a scan speed of 100 nm min^−1^ at room temperature. Slit widths with a nominal bandpass of 5 nm were used for both excitation and emission ray. A total of 0.5% *w*/*v* of the oil sample was dissolved in isooctane for analysis. Isooctane was used as a blank. The samples were assessed once per week for four weeks during accelerated oxidation of oils at 60 °C in the dark. Each run was a result of three accumulative scans. Experiments were performed in triplicate.

#### 2.2.7. Mayonnaise Preparation 

The preparation of mayonnaises was based on a previously reported work by Savaghebi et al. [29]. For the control sample, unmodified POO served as the oil phase, while modified oil with HTYR was used for the preparation of the fortified mayonnaise. The samples were stored under inert gas in screw-capped vials with Teflon at 28 °C, and their oxidative stability was evaluated once per month for five months. To extract the lipid phase from the emulsion systems, the procedure described by Lagunes-Galvez et al. [30] was followed to perform further analysis (CD and TBARS). Experiments were performed in triplicate.

#### 2.2.8. Statistical Analysis

All analyses were carried out in triplicate, in which the results were recorded as mean ± standard deviation. Student’s *t*−test, one-way ANOVA analysis and Tukey’s multiple comparison test were carried out using IBM SPSS Statistics version 21 (SPSS Inc., Chicago, IL, USA) to compare the mean values of each treatment and to determine the statistical significance (*p* < 0.05).

## 3. Results

### 3.1. Enzymatic Modification of Oil with Natural Antioxidants

**Figure 1:**
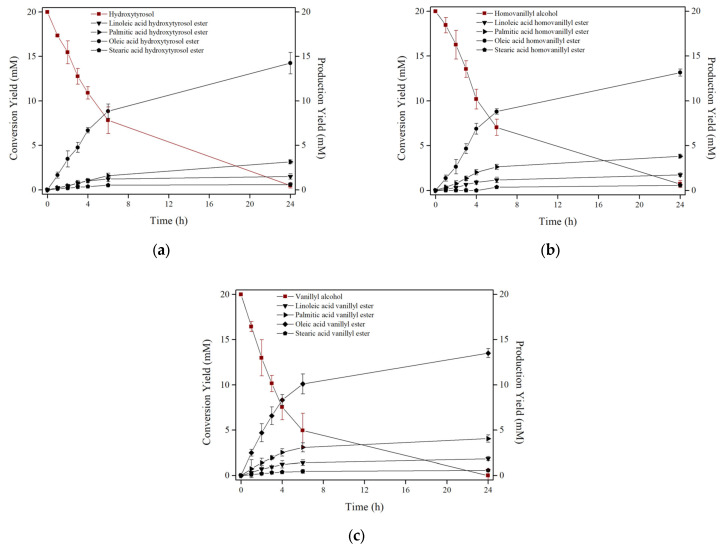
Time courses of enzymatic acylation of (**a**) HTYR, (**b**) HVA and (**c**) VA with POO in SFS by ZnOFe-TLL, showing the conversion yield of the substrates and the production yield of the lipophilic derivatives.

In the present study, we aimed to investigate the enzymatic acylation of phenolic compounds with a vegetable oil as a novel approach to produce natural lipophilic antioxidants/modified oils rich in structured phenolipids under benign reaction conditions to examine the phenomenon of rancidity, and the effect of the liposoluble products. HTYR, VA and HVA were exploited as the natural antioxidants (acyl acceptors), while POO served as the acylating agent in SFS. The fatty acid composition of POO was analysed by GC/MS, which revealed several saturated, monounsaturated and polyunsaturated fatty acids with oleic, palmitic, linoleic, and stearic acid as the more representative (Table S1). Lipase immobilized on the surface of zinc oxide-iron oxide nanoparticles was exploited as the biocatalyst, as reported before [16]. The progress of the reactions was monitored by HPLC-PDA. Figure 1a–c illustrates the conversion yield of the phenolic alcohols and the production yield of the main phenolipids. To identify the reaction products, several control reactions of the antioxidants with free fatty acids were conducted and their retention times were found. As expected, acylation increases the lipophilicity of the natural antioxidants. Therefore, phenolipids were eluted to longer retention times than the corresponding phenolic alcohols as recorded by the HPLC-PDA system. A typical chromatogram of HTYR esterification is presented in Figure S1. Moreover, as shown in Figure 1, the progress of the acylation reaction slightly differs between the three phenolic compounds. More specifically, the initial conversion rates for HTYR, VA and HVA were 0.20, 0.26 and 0.23 mM h^−1^ mg^−1^ nanobiocatalyst, respectively. Overall, the total yield after 24 h of reaction was approximately 98 ± 2% in any case. Milivojević et al. reported the enzymatic acylation of flavonoids with different natural oils in OSS reaching yields up to 90% after 92 h of incubation [22]. Ćorović et al. prepared L-ascorbyl esters exploiting different oil acyl donors in OSS with transesterification yields of up to 85.7% after 92 h of incubation [21]. Hence, the conversion yield and the incubation time for the preparation of lipophilic derivatives of natural antioxidant compounds depends on the acyl acceptor, the acyl donor, the reaction solvent, the temperature, and the lipase used [4,18].

To further characterize the novel phenolipids of its reaction, the samples were analysed by APCI-MS. In all cases, it could be noticed that the mass spectra depicted the molecular masses which correspond to [M+H]^+^ counterparts of monoesters of the three aromatic alcohols with the major fatty acids present in POO (oleic, palmitic, linoleic and stearic acid, Table S2). Figure S2 of the APCI-MS analysis ascertained the synthesis of phenolipids, which was in accordance with the results of HPLC-PDA. It is known that ester synthesis was initiated due to Le Chetelie’s thermodynamic principle; to reduce the excess of the acyl donor (POO), the reaction’s equilibrium shifts towards synthesis [31]. POO proved an excellent acyl donor for the enzymatic modification of different phenolic alcohols. These findings support the main goal of the study which was the preparation of modified oils rich in structured phenolipids/lipophilic antioxidant compounds in a SFS using an immobilized lipase and a low-cost edible oil as an acylating agent.

#### 3.1.1. Optimization of the Enzymatic Modification of Oil with HTYR 

The enzymatic acylation of HTYR with POO in SFS was used as the model reaction to optimize the reaction conditions such as the incubation temperature and the amount of biocatalyst. 

**Figure 2:**
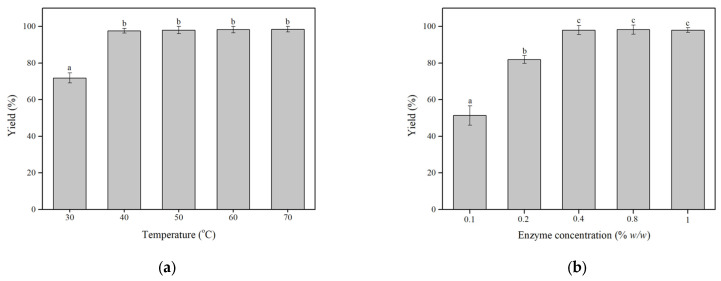
Effect of (**a**) temperature and (**b**) enzyme concentration on the enzymatic preparation of the modified oil with HTYR in SFS for 24 h at 180 rpm. Means with the same letter are not statistically different at *p* > 0.05 according to Tukey’s test.

Temperature is a key variable that affects the enzyme activity and stability. The acylation of HTYR by ZnOFe-TLL was carried out in the temperature range of 30–70 °C, and the obtained conversion yields are depicted in Figure 2a. As shown, with the advent of temperature from 30 to 40 °C, the yield of the reaction increased from 71.9 ± 2.9 to 97 ± 1.3%. However, the conversion yield did not show any statistically significant change with further increases in temperature (*p* > 0.05). These results indicated that the temperature of 40 °C was the optimum for the esterification of HTYR by ZnOFe-TLL achieving yields up to approximately 98%. Relevant studies have reported different optimal temperatures for the enzymatic acylation of phenolic compounds which is related to the biocatalyst (lipases), the reaction media, the substrates exploited, and other reaction conditions [18,22].

The enzyme concentration is another important variable that affects the effectiveness of the process as well as the overall application costs. Therefore, the influence of the enzyme concentration 0.1–1% (*w*/*w*) was evaluated in the optimal conditions (40 °C, 24 h). As shown in Figure 2b, as the enzyme concentration increased, the conversion yield linearly increased. More specifically, the conversion yield significantly increased from 51.4 ± 5.35 to 98%, when the enzyme concentration increased from 0.1 to 0.4 % (*w*/*w*). However, from 0.4 to 1% (*w*/*w*), the slight increase in the conversion yield was not statistically significant (*p* > 0.05). These findings indicated that 0.4% (*w*/*w*) of the enzyme was the optimum quantity to achieve the expected yield for the acylation of HTYR. Similar studies have also presented such a pattern indicating that equilibrium was reached [4]. Decreasing the enzyme amount required for a specific reaction while achieving high conversion yields is of paramount importance as far as the economical point of the application.

In the optimized experimental conditions, the yield of reaction with Lipozyme TL IM reached up to 97.3 ± 1.7 which is similar and not statistically different from the results obtained with ZnOFe-TLL. Hence, the green nanobiocatalytic system is comparable with industrial lipases.

#### 3.1.2. Operational Stability of the Nanobiocatalytic System

**Figure 3:**
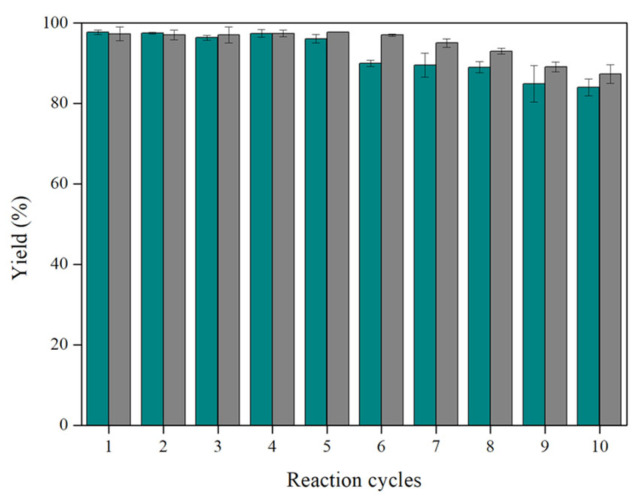
Operational stability of Lipozyme TL IM (grey bars) and ZnOFe-TLL (cyan bars) on the preparation of modified oil with HTYR using the optimal reaction conditions (40 °C, 0.4% *w*/*w*, 24 h, 180 rpm).

Concerning the widespread exploitation of immobilized lipases, the main goal is reutilization. In our work, ZnOFe-TLL was effectively recycled and operated in SFS for 240 h, achieving a yield of up to 84 ± 2.1% in the tenth successive cycle as for the acylation of HTYR, whereas Lipozyme TL IM showed a yield of up to 87.3 ± 2.3 in the tenth cycle, which is slightly higher compared to ZnOFe-TLL (Figure 3). The reusability of immobilized lipases has been rarely evaluated in relevant studies. Ćorović et al. assessed the operational stability of Novozyme 435 on the production of oil-based ascorbyl esters achieving yields of up to 73.5% in the tenth cycle [21]. Consequently, it is evident that the developed nanobiocatalytic system (ZnOFe-TLL) can be successfully reused under the optimized benign reaction conditions for the preparation of modified oils rich in structured phenolipids, accomplishing comparable operational stability with commercial lipases.

### 3.2. Antioxidant Evaluation of the Control and Modified Oils

**Figure 4:**
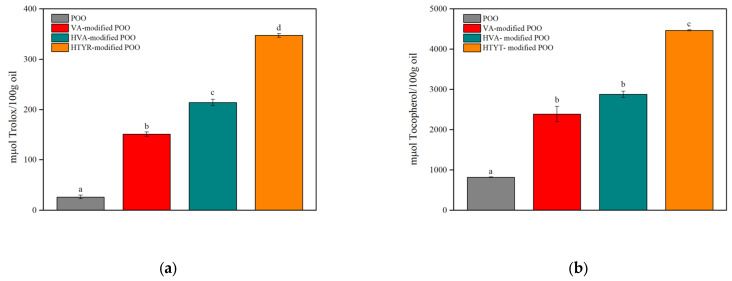
Antioxidant evaluation of the control oil and the modified oils after the enzymatic treatment by (**a**) the DPPH and (**b**) the CUPRAC assays. Means with the same letter are not statistically different at *p* > 0.05 according to Tukey’s test.

The antioxidant activity of the control oil and the modified oils was evaluated by the DPPH and the CUPRAC assays. As shown in Figure 4a,b, the antioxidant activity of POO was increased up to 12-times and up to 4.5-times after the enzymatic acylation with the phenolic compounds, as demonstrated by the DPPH and the CUPRAC assays, respectively. The unmodified POO exhibited negligible antioxidant activity. Interestingly, the enzymatic acylation significantly enhanced the antioxidant activity of POO. The antioxidant character of phenolics is preserved as their aromatic structure remains intact due to the enzymatic conjugation of fatty acids on their primary alcohol group. The modified oil with HTYR demonstrated the most favorable results. Among the modified oils with VA and HVA, the latter exhibited better antioxidant activity which according to the DPPH study was statistically significant (*p* < 0.05). The differences in the antioxidant activity observed between the modified oils prepared in this work were ascribed to the chemical structure of the three phenolic alcohols. HTYR has two aromatic hydroxyl groups (catecholic structure) in contrast to the one of HVA and VA, which subserve its ability to act as a stronger antioxidant [32,33]. Previous research has also demonstrated that the enzymatic process of lipophilization enhanced the antioxidant activity of the raw oils and acylating agents that were conjugated with ethyl ferulate [19]. Overall, the enzymatic acylation of the phenolic alcohols with POO resulted in novel modified oils rich in structured phenolipids with considerable antioxidant activity.

### 3.3. Oxidative Stability of the Control and Modified Oils

#### 3.3.1. Evaluation of the Primary and Secondary Oxidation Products

It is well-established that the incorporation of antioxidants in bulk oils or emulsion systems delays the onset of rancidity preserving the quality of the products. In our case, modified oils rich in structured phenolipids were prepared using natural aromatic alcohols, which exhibited enhanced antioxidant activity compared to the control oil. Therefore, the objective of this work was also to evaluate the oxidative stability of the enzymatically prepared oils under thermal treatment. 

**Figure 5:**
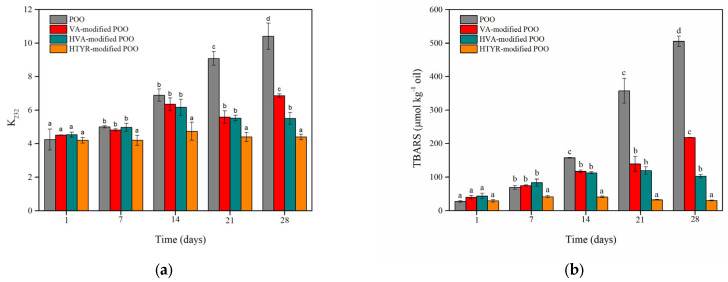
Conjugated diene (**a**) and Thiobarbituric acid reactive substance (**b**) formation analysis in the control oil and the modified oils under thermal treatment at 60 °C for 28 days. Means with the same letter are not statistically different at *p* > 0.05 according to Tukey’s test.

Accelerated oxidation of oils at 60 °C leads to precocious rancidity [34]. Conjugated dienes emerge from the rearrangement of the double bonds of unsaturated fatty acids. An increase in the conjugated dienes expressed as specific extinction coefficient values is a strong indication of the primary oxidation stage [35]. As shown in Figure 5a, the formation of conjugated dienes was monitored for a period of 28 days. At the first and second weeks of incubation, the modified oil with HTYR had a significant difference in the *K_232_* value which remained practically constant compared to the tested oils. Moreover, it was evident from the third week of incubation that the enzymatically prepared oils significantly inhibited the formation of the primary oxidation products in contrast to the control oil. The same trend was also apparent in the fourth week of incubation. Specifically, the *K_232_* values of POO, VA-modified POO, HVA-modified POO and HTYR-modified POO were 10.41 ± 0.78, 6.86 ± 0.11, 5.31 ± 0.35 and 4.4 ± 0.17, respectively. Consequently, the enzymatic treatment of POO with natural aromatic alcohols profoundly enhances the oxidative stability of the oil to the point of suppressing the production of primary oxidation compounds. Furthermore, the differences between the control oil and the modified oils were statistically significant (*p* < 0.05). The modified oil with HTYR presents the most evident stabilizing effect, followed by the modified oil with HVA and, finally, the modified oil with VA. These findings were consistent with the results of the antioxidant evaluation. Natalia et al. also evaluated the CDs formed within an emulsion that included fatty acid vanillyl esters [4]. The CDs formation rate was decreased in comparison to the control sample, implying that the enzymatically derived VA esters had a significant impact on the prevention of oxidation. The same effect was noticed by incorporating enzymatically derived rutin esters in sardine oil. The CDs formation was suppressed during storage at 37 °C for 20 days [36].

Among the primary oxidation products are the highly reactive hydroxyperoxides. Hydroxyperoxides rapidly decompose to secondary oxidative products (aldehydes, ketones, alkenals, alkadienals). TBARS is the most common method used to evaluate the second stage of oxidation. TBA reacts with aldehydes, ketones, and alkadienals, with malonaldehyde being the most representative reactive compound [18,37]. Several secondary products are derived from the degradation of oleic and linoleic acid [27,34]. As shown in Figure 5b, the formation of TBARS was monitored for a period of 28 days. After two weeks of incubation, the TBARS content of the tested oils was increased except for the modified oil with HTYR. From the third week of incubation at 60 °C, it was noticeable that the enzymatically prepared oils significantly inhibited the formation of the secondary oxidation products in contrast to the control oil. This observation was also evident in the fourth week of incubation. The TBARS contents of POO, VA-modified POO, HVA-modified POO and HTYR-modified POO were 505.25 ± 15.63, 217.95 ± 1.18, 102.79 ± 5.24 and 30.41 ± 1.14 μmol kg^−1^ of oil, respectively. Consequently, the enzymatic treatment of POO with natural aromatic alcohols remarkably enhanced the oxidative stability of the oil to the point of suppressing rancidity. Furthermore, from the second to the fourth week of incubation, the differences between the control oil and the modified oils were statistically significant (*p* < 0.05). These findings were consistent with the results of the antioxidant evaluation and the CD test. Overall, the modified oil with HTYR demonstrated a significantly stabilizing effect, inhibiting completely pomace olive oil’s degradation. Rutin fatty acid esters also delayed the progress of secondary oxidative product formation in sardine oil [36]. Consequently, the enzymatically derived fatty acid esters of phenolics and flavonoids can hinder the rate of oxidation in edible vegetable oils.

#### 3.3.2. Evaluation of Volatile Oxidative Products by SPME-GC/MS

Secondary oxidative products formed during thermal treatment are mostly volatile compounds to which the unpleasant aroma of oxidized oils is ascribed [27]. SPME-GC/MS analysis was conducted to gain a deeper insight into the formation of specific secondary products. As shown in Figure S3, several volatile compounds were identified. Among them, 2-octenal and nonanal were selected as model compounds to evaluate the rate of oxidation. 2-Octenal derives from the decomposition of linoleic acid, whereas nonanal derives from the degradation of oleic acid [27,34]. As depicted in Figure S4a,b, the oxidation of control oil proceeds more rapidly in contrast to that of the modified oils. During the second and third weeks of incubation, these findings were more evident. 2-Octenal and nonanal content in the control oil was up to 117.6 times and 10.3 times greater, respectively, compared to the modified oils. Overall, the enzymatically modified oils with the phenolic compounds decreased the rate of secondary oxidative product formation. Difonzo et al. also reported that the incorporation of an olive leaf extract suppressed the production of volatile oxidative compounds in baked snacks, improving the sensory quality of the products [38].

#### 3.3.3. Fluorescence Spectroscopy

**Figure 6:**
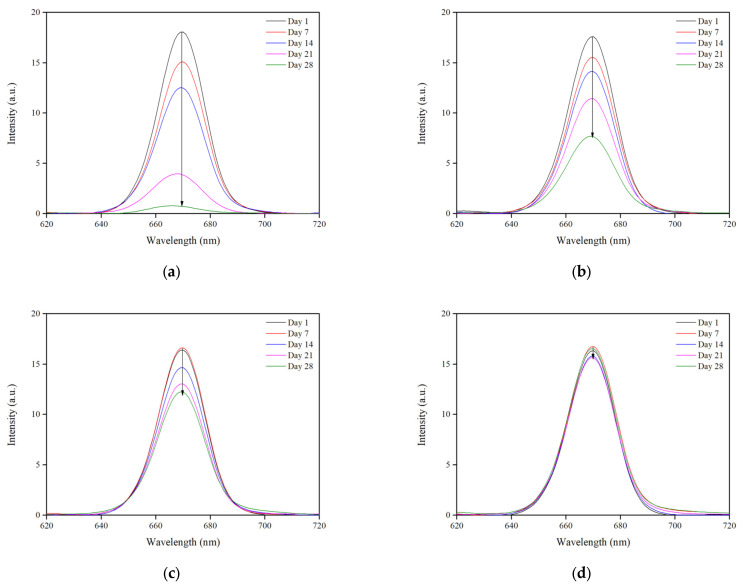
Fluorescence emission spectra of the oil samples: (**a**) POO, (**b**) VA-modified POO, (**c**) HVA-modified POO and (**d**) HTYR-modified POO.

Fluorescence spectroscopy was also used as a tool to access the adulteration of the control and the modified oils. The decrease in the fluorescence peak at 670 nm after excitation of oil samples at 400 nm is ascribed to oil pigments, specifically chlorophylls, which undergo deterioration under thermal treatment. Therefore, the fluorescence emission peak at 670 nm is a good indicator of the degradation of oils [39,40]. As shown in Figure 6, the fluorescence peak of the control oil decreased 18-fold after 28 days at 60 °C, whereas the fluorescence peaks of the modified oils with HVA and HTYR were remarkably preserved. The modified oil with VA exhibited moderate results (2.5-fold decrease), while the modified oil with HTYR presented the most evident positive effect. These observations agree with those of Hao et al. concerning the effect of heat on the oil pigments and the importance of using antioxidants to preserve the quality of the products [39].

### 3.4. Oxidative Stability of Mayonnaise Formulations

Mayonnaise is the most common sauce worldwide. The main constituent is oil; therefore, mayonnaise is highly susceptible to autoxidation due to extrinsic and intrinsic factors. The efficacy of the modified oil with HTYR to prevent or retard the production of primary and secondary oxidative products in mayonnaise sauces was estimated by CD and TBARS assays, respectively. Therefore, two different mayonnaises were prepared; the control mayonnaise using POO and the fortified mayonnaise containing enzymatically modified POO with HTYR. The oxidative deterioration of the sauces was monitored for a period of five months under storage at 28 °C in the dark. 

**Figure 7:**
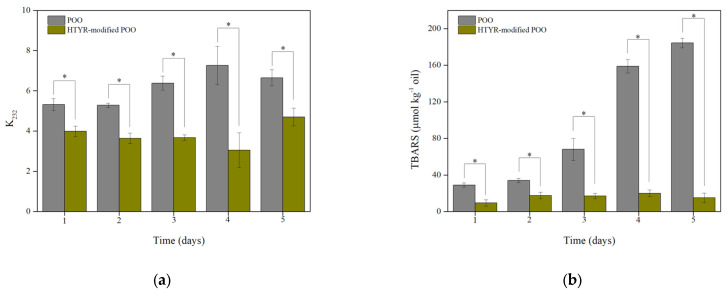
Conjugated diene (**a**) and Thiobarbituric acid reactive substance (**b**) formation analysis in the control and fortified mayonnaises under storage at 28 °C for 5 months. Asterisks indicate statistically significant differences according to Student’s *t*−test (*p* < 0.05).

As depicted in Figure 7a, the *K_232_* value of the control mayonnaise was increased from the first month of storage in contrast to the fortified mayonnaise. After four months of storage, the *K_232_* value of the fortified mayonnaise remained constant, whereas the *K_232_* value of the control had an increment tendency. These differences were also statistically significant (*p* < 0.05), highlighting the potent antioxidant capacity of the novel modified oil with HTYR.

The TBARS content also demonstrated an upward trend. As shown in Figure 7b, during the first and second months of storage, the TBARS content increased slightly in the mayonnaise samples; however, the difference between the control and fortified sauce was statistically significant (*p* < 0.05). From the third month, it is noticeable that the formation of secondary oxidative products in the control mayonnaise rapidly progressed. 

The results of lipid peroxidation in mayonnaise samples were in agreement with those of the neat oils. These observations are a reliable indication for the exploitation of enzymatically modified oils into emulsion systems. Many studies have underlined the protective effect of plant antioxidants on the stability of mayonnaise sauces, as interpreted by similar techniques. Lipophilic phenolic and flavonoid compounds provably suppress the oxidative cascade reactions, preserving the quality of sauces [41,42]. In our work, a novel modified oil rich in structured HTYR phenolipids was used as the lipid phase of mayonnaise formulation, exhibiting a strong protective effect, although further organoleptic screening is required. 

## 4. Conclusions

In this study, novel modified oils rich in structured phenolipids were prepared by one-pot lipophilization of phenolic compounds with POO. ZnOFe-TLL, a green nanobiocatalytic system, was used as the biocatalyst. The optimum reaction conditions were 40 °C, 0.4% *w*/*w* of immobilized lipase and 24 h of incubation. Under the optimized conditions, the conversion yield reached up to 98%, and the immobilized lipase was reused up to ten times. 

Among the new oils and the control, the modified oil with HTYR exerted the highest antioxidant activity. Furthermore, the oxidative stability of POO was significantly enhanced after the enzymatic modification, with the case of the HTYR-modified oil as the more representative. Finally, the stability of a fortified mayonnaise was compared with that of a control mayonnaise at 28 °C for 5 months, and the results seem to open up new strategies regarding the application of the enriched oils in food systems in order to retard rancidity.

The scientific contribution of this work lies in the development of a biocatalytic process that utilizes a novel, reusable nanobiocatalyst for the preparation of lipophilic derivatives of natural antioxidants in SFS, exploiting a cheap bioresource such as POO as an acylating agent. Moreover, the whole procedure was conducted in benign conditions, eliminating the overall application costs. It is worth noting that the use of lipases is of utmost importance for industrial applications with a scope to replace traditional chemical synthesis and comfort with the principles of green chemistry. Enzymatically modified oils with lipophilic antioxidants could have potential application in the food industry as more stable alternatives of common vegetable oils.

## Data Availability

Not applicable.

## Supplementary Materials

The following supporting information can be downloaded at: https://www.mdpi.com/article/10.3390/biom13071034/s1, Table S1: Fatty acid composition (%) of Pomace Olive Oil, Figure S1: HPLC chromatogram of the enzymatic acylation of HTYR with POO monitored at 280 nm. The peak at 3.05 min corresponds to HTYR and the peaks eluted from 18 to 24.5 min correspond to the major phenolipids (1: linoleic acid HTYR ester, 2: palmitic acid HTYR ester, 3: oleic acid HTYR ester, 4: stearic acid HTYR ester), Figure S2: Mass spectra of lipophilic derivatives of natural antioxidants acquired in positive source of atmospheric pressure chemical ionization (APCI), Table S2: Chemical structure of lipophilic derivatives of natural antioxidants and their expected masses (m/z), Figure S3: SPME-GC/MS chromatogram of the volatile secondary oxidative products formed during thermal treatment at 60 °C. The peak at 15.4 min corresponds to 2-octenal (similarity index: 92%) and the peak at 16.5 min to nonanal (similarity index: 97%), Figure S4: Monitoring the secondary volatile oxidative products: (a) 2-Octenal and (b) Nonanal in the control and modified oils under thermal treatment at 60 °C for 21 days.

## References

[1] Atallah E., Kwapinski W., Ahmad M.N., Leahy J.J., Al-Muhtaseb A.H., Zeaiter J. (2019). Hydrothermal Carbonization of Olive Mill Wastewater: Liquid Phase Product Analysis. J. Environ. Chem. Eng..

[2] Bernini R., Carastro I., Santoni F., Clemente M. (2019). Synthesis of Lipophilic Esters of Tyrosol, Homovanillyl Alcohol and Hydroxytyrosol. Antioxidants.

[3] Chatzikonstantinou A.V., Giannakopoulou A., Spyrou S., Simos Y.V., Kontogianni V.G., Peschos D., Katapodis P., Polydera A.C., Stamatis H. (2022). Production of Hydroxytyrosol Rich Extract from Olea Europaea Leaf with Enhanced Biological Activity Using Immobilized Enzyme Reactors. Environ. Sci. Pollut. Res..

[4] Natalia A., Kim S.J., Kim H.K. (2016). Antioxidant and Antibacterial Activity of Fatty Acid Vanillyl Ester Produced by Proteus Vulgaris K80 Lipase-Mediated Transesterification. J. Mol. Catal. B Enzym..

[5] Serreli G., Deiana M. (2018). Biological Relevance of Extra Virgin Olive Oil Polyphenols Metabolites. Antioxidants.

[6] Achmon Y., Fishman A. (2015). The Antioxidant Hydroxytyrosol: Biotechnological Production Challenges and Opportunities. Appl. Microbiol. Biotechnol..

[7] Liu L., Jin C., Zhang Y. (2014). Lipophilic Phenolic Compounds (Lipo-PCs): Emerging Antioxidants Applied in Lipid Systems. RSC Adv..

[8] González-Rámila S., Mateos R., García-Cordero J., Seguido M.A., Bravo-Clemente L., Sarriá B. (2022). Olive Pomace Oil versus High Oleic Sunflower Oil and Sunflower Oil: A Comparative Study in Healthy and Cardiovascular Risk Humans. Foods.

[9] Mateos R., Sarria B., Bravo L. (2019). Nutritional and Other Health Properties of Olive Pomace Oil. Crit. Rev. Food Sci. Nutr..

[10] Serra A., Conte G., Giovannetti M., Casarosa L., Agnolucci M., Ciucci F., Palla M., Bulleri E., Cappucci A., Servili M. (2018). Olive Pomace in Diet Limits Lipid Peroxidation of Sausages from Cinta Senese Swine. Eur. J. Lipid Sci. Technol..

[11] Maqsood S., Benjakul S., Abushelaibi A., Alam A. (2014). Phenolic Compounds and Plant Phenolic Extracts as Natural Antioxidants in Prevention of Lipid Oxidation in Seafood: A Detailed Review. Compr. Rev. Food Sci. Food Saf..

[12] Tarapoulouzi M., Agriopoulou S., Koidis A., Proestos C., Enshasy H.A.E., Varzakas T. (2022). Recent Advances in Analytical Methods for the Detection of Olive Oil Oxidation Status during Storage along with Chemometrics, Authenticity and Fraud Studies. Biomolecules.

[13] Arzola-Rodríguez S.I., Muñoz-Castellanos L.N., López-Camarillo C., Salas E. (2022). Phenolipids, Amphipilic Phenolic Antioxidants with Modified Properties and Their Spectrum of Applications in Development: A Review. Biomolecules.

[14] Xanthakis E., Theodosiou E., Magkouta S., Stamatis H., Loutrari H., Roussos C., Kolisis F. (2010). Enzymatic Transformation of Flavonoids and Terpenoids: Structural and Functional Diversity of the Novel Derivatives. Pure Appl. Chem..

[15] Zieniuk B., Groborz K., Wołoszynowska M., Ratusz K., Białecka-florjańczyk E., Fabiszewska A. (2021). Enzymatic Synthesis of Lipophilic Esters of Phenolic Compounds, Evaluation of Their Antioxidant Activity and Effect on the Oxidative Stability of Selected Oils. Biomolecules.

[16] Fotiadou R., Chatzikonstantinou A.V., Hammami M.A., Chalmpes N., Moschovas D., Spyrou K., Polydera A.C., Avgeropoulos A., Gournis D., Stamatis H. (2021). Green Synthesized Magnetic Nanoparticles as Effective Nanosupport for the Immobilization of Lipase: Application for the Synthesis of Lipophenols. Nanomaterials.

[17] Fotiadou R., Patila M., Hammami M.A., Enotiadis A., Moschovas D., Tsirka K., Spyrou K., Giannelis E.P., Avgeropoulos A., Paipetis A. (2019). Development of Effective Lipase-Hybrid Nanoflowers Enriched with Carbon and Magnetic Nanomaterials for Biocatalytic Transformations. Nanomaterials.

[18] Sun S., Zhu S., Bi Y. (2014). Solvent-Free Enzymatic Synthesis of Feruloylated Structured Lipids by the Transesterification of Ethyl Ferulate with Castor Oil. Food Chem..

[19] Zhang H., Zheng M., Shi J., Tang H., Deng Q., Huang F., Luo D. (2018). Enzymatic Preparation of “Functional Oil” Rich in Feruloylated Structured Lipids with Solvent-Free Ultrasound Pretreatment. Food Chem..

[20] European Union (1991). Commission Regulation (EEC) No. 2568/91.

[21] Ćorović M., Milivojević A., Simović M., Banjanac K., Pjanović R., Bezbradica D. (2020). Enzymatically Derived Oil-Based L-Ascorbyl Esters: Synthesis, Antioxidant Properties and Controlled Release from Cosmetic Formulations. Sustain. Chem. Pharm..

[22] Milivojević A.D., Ćorović M.M., Simović M.B., Banjanac K.M., Blagojević S.N., Pjanović R.V., Bezbradica D.I. (2019). Novel Approach for Flavonoid Esters Production: Statistically Optimized Enzymatic Synthesis Using Natural Oils and Application in Cosmetics. Ind. Eng. Chem. Res..

[23] Harris C.S., Burt A.J., Saleem A., Le P.M., Martineau L.C., Haddad P.S., Bennett S.A.L., Arnason J.T. (2007). A Single HPLC-PAD-APCI/MS Method for the Quantitative Comparison of Phenolic Compounds Found in Leaf, Stem, Root and Fruit Extracts of Vaccinium Angustifolium. Phytochem. Anal..

[24] Tose L., Silva S., Barros E., Souza L., Pinto F., Palomino D., Freitas J., Thompson C., Vaz B., Lacerda V. (2019). APCI(+)FT-ICR MS Analysis of Hydrocarbons Using Isooctane as Ionizing Reagent—A Comparison with HTGC-FID, GC×GC-MS and NMR. J. Braz. Chem. Soc..

[25] Özyürek M., Güçlü K., Tütem E., Bakan K.S., Erçaǧ E., Esin Çelik S., Baki S., Yildiz L., Karaman Ş., Apak R. (2011). A Comprehensive Review of CUPRAC Methodology. Anal. Methods.

[26] Zeb A., Ullah F. (2016). A Simple Spectrophotometric Method for the Determination of Thiobarbituric Acid Reactive Substances in Fried Fast Foods. J. Anal. Methods Chem..

[27] Beltrán A., Ramos M., Grané N., Martín M.L., Garrigós M.C. (2011). Monitoring the Oxidation of Almond Oils by HS-SPME-GC-MS and ATR-FTIR: Application of Volatile Compounds Determination to Cultivar Authenticity. Food Chem..

[28] Grebenteuch S., Kroh L.W., Drusch S., Rohn S. (2021). Formation of Secondary and Tertiary Volatile Compounds Resulting from the Lipid Oxidation of Rapeseed Oil. Foods.

[29] Savaghebi D., Ghaderi-Ghahfarokhi M., Barzegar M. (2021). Encapsulation of *Sargassum Boveanum* Algae Extract in Nano-Liposomes: Application in Functional Mayonnaise Production. Food Bioprocess Technol..

[30] Lagunes-Galvez L., Cuvelier M.-E., Ordonnaud C., Berset C. (2002). OXIDATIVE STABILITY OF SOME MAYONNAISE FORMULATIONS DURING STORAGE AND DAYLIGHT IRRADIATION. J. Food Lipids.

[31] Roby M.H., Allouche A., Dahdou L., De Castro V.C., Alves Da Silva P.H., Targino B.N., Huguet M., Paris C., Chrétien F., Guéant R.M. (2015). Enzymatic Production of Bioactive Docosahexaenoic Acid Phenolic Ester. Food Chem..

[32] Bouguerra Neji S., Bouaziz M. (2022). Production of Biologically Active Hydroxytyrosol Rich Extract: Via Catalytic Conversion of Tyrosol. RSC Adv..

[33] Charlton N.C., Mastyugin M., Török B., Török M. (2023). Structural Features of Small Molecule Antioxidants and Strategic Modifications to Improve Potential Bioactivity. Molecules.

[34] Domínguez R., Pateiro M., Gagaoua M., Barba F.J., Zhang W., Lorenzo J.M. (2019). A Comprehensive Review on Lipid Oxidation in Meat and Meat Products. Antioxidants.

[35] Gomes I.A., Lindenblatt C.T., Masson L.M.P., Gomes F.D.S., Freitas-Silva O., Silva J.P.L. (2016). Effect of oregano essential oil on oxidative stability of low-acid mayonnaise. IOSR J. Pharm..

[36] Chandrasekar V., Prasanna D.B., Regupathi I. (2018). Effectiveness of Rutin and Its Lipophilic Ester in Improving Oxidative Stability of Sardine Oil Containing Trace Water. Int. J. Food Sci. Technol..

[37] Koh E., Ryu D., Surh J. (2015). Ratio of Malondialdehyde to Hydroperoxides and Color Change as an Index of Thermal Oxidation of Linoleic Acid and Linolenic Acid. J. Food. Process. Preserv..

[38] Difonzo G., Pasqualone A., Silletti R., Cosmai L., Summo C., Paradiso V.M., Caponio F. (2018). Use of Olive Leaf Extract to Reduce Lipid Oxidation of Baked Snacks. Food Res. Int..

[39] Hao S., Zhu L., Sui R., Zuo M., Luo N., Shi J., Zhang W., He X., Chen Z. (2019). Identification and Quantification of Vegetable Oil Adulteration with Waste Frying Oil by Laser-Induced Fluorescence Spectroscopy. OSA Contin..

[40] Kongbonga Y.G.M., Ghalila H., Onana M.B., Majdi Y., Lakhdar Z.B., Mezlini H., Sevestre-Ghalila S. (2011). Characterization of Vegetable Oils by Fluorescence Spectroscopy. Food Nutr. Sci..

[41] Alizadeh L., Abdolmaleki K., Nayebzadeh K., Shahin R. (2019). Effects of Tocopherol, Rosemary Essential Oil and Ferulago Angulata Extract on Oxidative Stability of Mayonnaise during Its Shelf Life: A Comparative Study. Food Chem..

[42] Ozdemir N., Kantekin-Erdogan M.N., Tat T., Tekin A. (2018). Effect of Black Cumin Oil on the Oxidative Stability and Sensory Characteristics of Mayonnaise. J. Food Sci. Technol..

