# Supplementary material for: Enzymatic Modification of Pomace Olive Oil with Natural Antioxidants: Effect on Oxidative Stability
Source: Biomolecules. 2023 Jun 23;13(7):1034. doi: 10.3390/biom13071034 (PMC10377084; doi:10.3390/biom13071034)
Supplement: Supplementary file 1 [file biomolecules-13-01034-s001.zip › biomolecules-2459799-supplementary.pdf]

Supplementary Material

Enzymatic Modification of Pomace Olive Oil with Natural Antioxidants: Effect on Oxidative Stability

Renia Fotiadou [1], Dimitrios Lefas [1], Despina Vougiouklaki [2], Aliki Tsakni [2], Dimitra Houhoula [2] and Haralambos Stamatis [1,*]

[1] Laboratory of Biotechnology, Department of Biological Applications and Technologies, University of Ioannina, 45110 Ioannina, Greece
[2] Department of Food Science and Technology, University of West Attica, 12243 Athens, Greece
* Correspondence: hstamati@uoi.gr

Table S1. Fatty acid composition (%) of Pomace Olive Oil

Fatty Acids	Pomace Olive Oil
Palmitoleic acid (C16:1)	0.66 ± 0.06
Palmitic acid (C16:0)	13.25 ± 0.11
trans-13-Octadecenoic acid (C19:1)	0.02 ± 0.02
Linoleic acid (C18:2)	9.80 ± 0.26
Oleic acid (C18:1)	72.67 ± 0.71
Stearic acid (C18:0)	3.02 ± 0.10
11-Eicosenoic acid (C21:1)	0.13 ± 0.04
Arachidic acid (20:0)	0.35 ± 0.07
Behenic acid (22:0)	0.05 ± 0.02
**Σ SFA**	16.68 ± 0.07
**Σ MUFA**	73.49 ± 0.21
**Σ PUFA**	9.8 ± 0.26

Values are represented as the means of three replicates ± standard deviation;
SFA – saturated fatty acid; MUFA – monounsaturated fatty acid;
PUFA – polyunsaturated fatty acid.

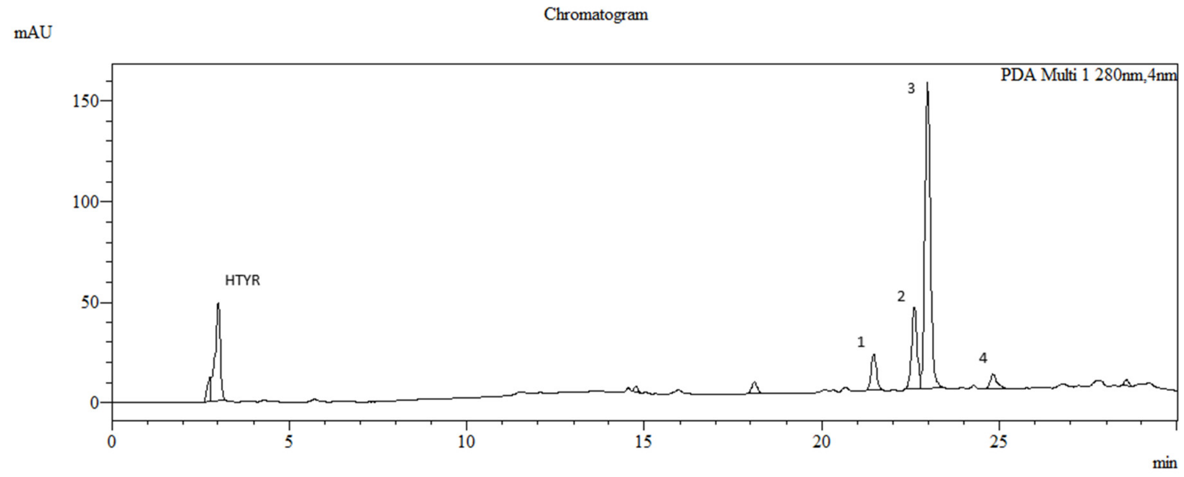

Figure S1. HPLC chromatogram of the enzymatic acylation of HTYR with POO monitored at 280 nm. The peak at 3.05 min corresponds to HTYR and the peaks eluted from 18 to 24.5 min correspond to the major phenolipids (1: linoleic acid HTYR ester, 2: palmitic acid HTYR ester, 3: oleic acid HTYR ester, 4: stearic acid HTYR ester).

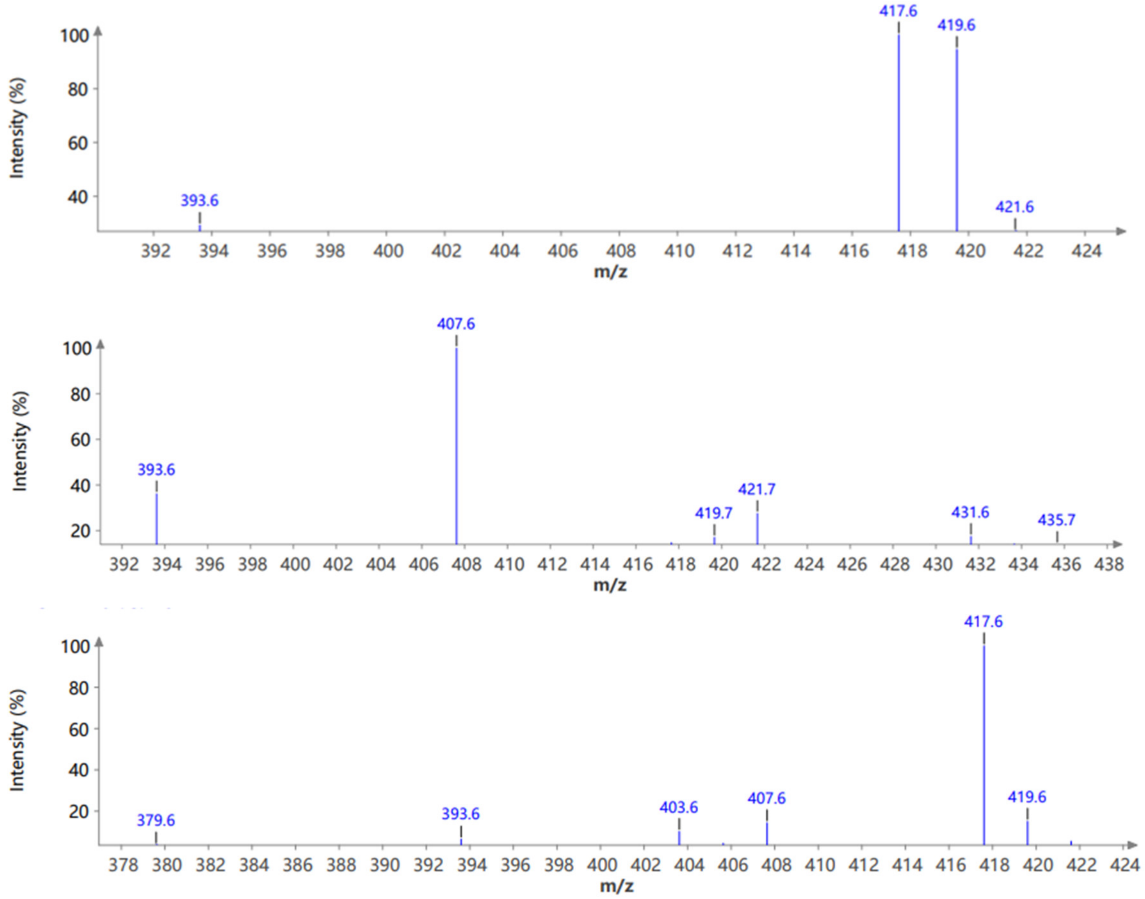

Figure S2. Mass spectra of lipophilic derivatives of natural antioxidants acquired in positive source of atmospheric pressure chemical ionization (APCI).

Table S2. Chemical structure of lipophilic derivatives of natural antioxidants and their expected masses (m/z).

Phenolic Compound	Acyl group	m/z[a]
Hydroxytyrosol	R= $CH_3(CH_2)_4CH{=}CHCH_2CH{=}CH(CH_2)_7$-[b]	417.6
	R=$CH_3(CH_2)_{14}$-[c]	393.6
	R=$CH_3(CH_2)_7CH{=}CH(CH_2)_7$-[d]	419.6
	R=$CH_3(CH_2)_{16}$-[e]	421.6
Homovanillyl alcohol	R= $CH_3(CH_2)_4CH{=}CHCH_2CH{=}CH(CH_2)_7$-[b]	431.6
	R=$CH_3(CH_2)_{14}$-[c]	407.6
	R=$CH_3(CH_2)_7CH{=}CH(CH_2)_7$-[d]	433.6
	R=$CH_3(CH_2)_{16}$-[e]	435.6
Vanillyl alcohol	R= $CH_3(CH_2)_4CH{=}CHCH_2CH{=}CH(CH_2)_7$-[b]	417.6
	R=$CH_3(CH_2)_{14}$-[c]	393.6
	R=$CH_3(CH_2)_7CH{=}CH(CH_2)_7$-[d]	419.6
	R=$CH_3(CH_2)_{16}$-[e]	421.6

[a] $[M+H]^+$
[b] Linoleic acid phenolic ester
[c] Palmitic acid phenolic ester
[d] Oleic acid phenolic ester
[e] Stearic acid phenolic ester

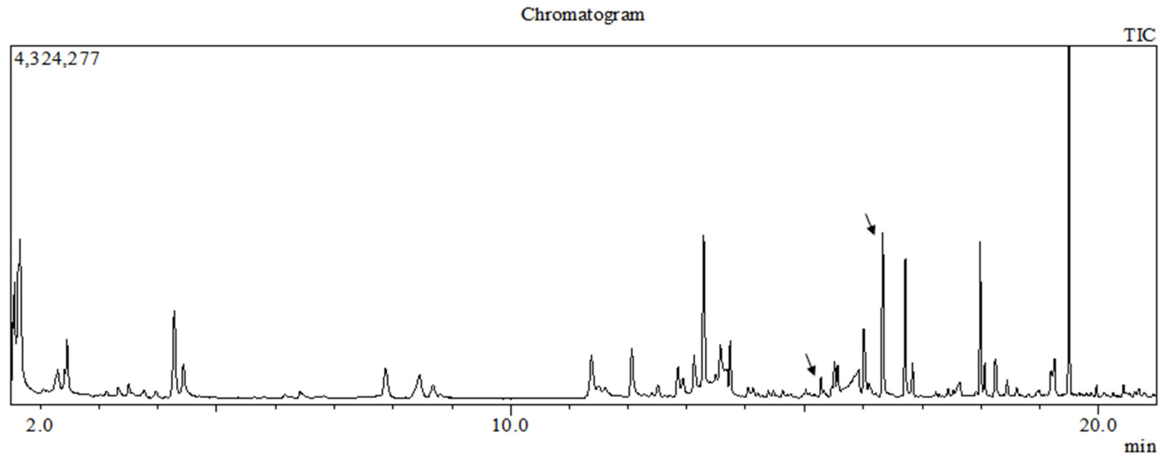

Figure S3. SPME-GC/MS chromatogram of volatile secondary oxidative products formed during thermal treatment at 60 °C. The peak at 15.4 min corresponds to 2-octenal (similarity index: 92%) and the peak at 16.5 min to nonanal (similarity index: 97%).

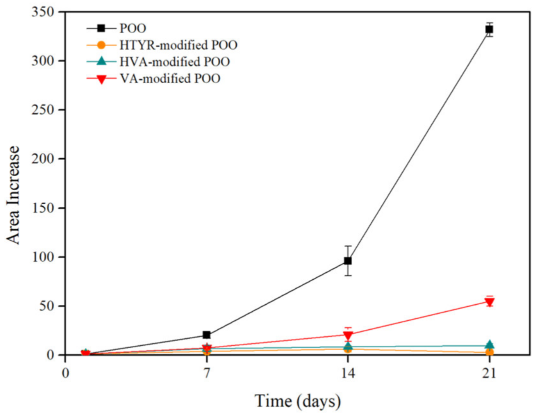

(**a**)

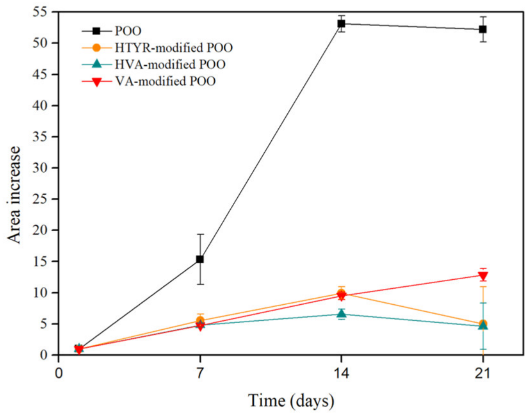

(**b**)

Figure S4. Monitoring the secondary volatile oxidative products: (a) 2-Octenal and (b) Nonanal in the control and modified oils under thermal treatment at 60 ºC for 21 days.
